# Single-Center Experience With the Bare p48MW Low-Profile Flow Diverter and Its Hydrophilically Covered Version for Treatment of Bifurcation Aneurysms in Distal Segments of the Anterior and Posterior Circulation

**DOI:** 10.3389/fneur.2020.01050

**Published:** 2020-09-23

**Authors:** Stefan Schob, Monika Kläver, Cindy Richter, Cordula Scherlach, Jens Maybaum, Simone Mucha, Marie-Sophie Schüngel, Karl Titus Hoffmann, Ulf Quaeschling

**Affiliations:** Abteilung für Neuroradiologie, Universitätsklinikum Leipzig, Leipzig, Germany

**Keywords:** flow diversion, p48MW, small cerebral vessels, cerebral aneurysm, reduced platelet function inhibition

## Abstract

**Background and Purpose:** Flow diversion has profoundly changed the way aneurysms are treated. However, it conventionally requires dual antiplatelet medication and has yet been considered off-label use in the posterior circulation or within peripheral vessels of the anterior circulation. Here, we report our experience with the p48MW/p48MW hydrophilic coating (HPC) in the anterior and posterior circulation. This novel low-profile flow diverter is specifically designed for treatment of small peripheral vessels, and the p48MW HPC has an anti-thrombotic polymer coating, which allows application of a single antiplatelet function medication in conditions that expectably require further surgery.

**Materials and Methods:** Thirty-two patients were prospectively included. Twenty-six treatments were performed with one flow diverter, four required two overlapping flow diverters, one case demanded three overlapping flow diverters, and in one case, extensive dissecting aneurysm telescoping with eight flow diverters was necessary. Twenty-two complex bifurcation aneurysms were treated. Three months' follow-up was available for 14 patients.

**Results:** Deployment was uneventful in all cases. In four cases, undersizing was unavoidable and resulted in significant shortening of the flow diverter, which demanded implantation of further flow diverters to sufficiently treat the target aneurysm. Three flow diverters required balloon angioplasty for optimal wall approximation. All parent vessels remained patent. Available 3-month follow-up studies showed decreased influx or delayed washout in all aneurysms; none was occluded completely. There were no device-related clinical complications.

**Conclusions:** Implantation of the p48MW/p48MW HPC is safe and effective for treatment of distally located cerebral aneurysms. Considering the reported rates of ischemic complications associated with flow diversion of complex bifurcation aneurysms, the p48MW/p48MW HPC potentially provides increased safety for complex bifurcation aneurysms in the anterior and posterior circulation.

## Introduction

Flow diverter stents (FDSs) have significantly improved endovascular treatment of challenging intracranial aneurysms in recent years (1, 2). A number of earlier studies reported good safety and efficacy in reviewing short-term follow-ups (3–5), which has recently been substantiated by reports of excellent long-term outcomes after implantation of the Pipeline embolization device (PED, Medtronic, Dublin, Ireland) (6) and the p64 (phenox, Bochum, Germany) (7).

In general, flow diversion is based on two synergistic mechanisms working in sequential order. Immediately after implantation into the aneurysmal segment, the densely woven mesh of the flow diverter reduces aneurysmal inflow and causes redirection of the bloodstream along the longitudinal axis of the parent vessel (8). Then stagnation of blood flow within the aneurysm induces progressive intra-aneurysmal thrombosis. A combination of the latter together with the cessation of flow through the aneurysmal orifice promotes the formation of a neo-intima along the stable scaffold of the device, which eventually embeds the FDSs into the vessel wall and permanently excludes the aneurysm from the circulation (9).

In the United States, implantation of FDSs for aneurysm treatment in the anterior circulation has been approved for use up to the terminus of the internal carotid artery (ICA) (2). However, off-label use of FDSs is increasingly performed on a regular basis (10, 11), and a growing body of evidence suggests its safety and efficacy for aneurysms in the posterior circulation and for distal segments of the circle of Willis (12–14).

Currently, a number of second-generation FDSs have been made available for endovascular surgeons (15). However, a significant drawback of treatment with most FDSs is the requirement of large microcatheters (0.027–0.033 in.) for delivery, which are hardly suitable for smooth and atraumatic navigation in elongated and peripheral intracranial vessels. The p48MW (phenox GmbH, Bochum, Germany) is a novel low-profile FDS, specifically designed for the treatment of small cerebral arteries ranging from 1.5 to 3 mm in diameter, which allows the deployment *via* a 0.021 in. inner diameter microcatheter and provides enhanced stability during implantation through its individually movable distal wire. Additionally, the p48MW has recently been made available with a hydrophilic coating (p48MW HPC), which facilitates the use of a less rigorous antiplatelet function regimen under circumstances where common dual platelet function inhibition cannot be applied (16, 17).

So far, the experience with low-profile flow diverters, including the p48MW, is limited (18). As a consequence, our study aims to report our single-center experience with prospectively collected cases of aneurysm treatment employing the p48MW and p48MW HPC in un-ruptured and ruptured intracranial aneurysms.

## Materials and Methods

### Ethics Committee Approval

Our study—systematically reviewing cases of p48MW implantations starting in January 2019—was approved by the institutional ethics committee [local institutional review board (IRB) nr. AZ 208-15-0010062015]. Informed consent of each patient regarding the scientific use of radiological and clinical data was obtained in writing from either the patient or his/her legal representative.

### Patients and Treatments

Our radiological information system was reviewed for FDS treatments using the p48MW. A total of 32 patients were identified (12 male and 20 female patients, mean age of 59 years, ranging from 34 to 85 years) Table 1 summarizes the relevant information of all included patients.

**Table 1:** Relevant information of all included patients.

**Patient**	**Age**	**Location**	**Previous SAH**	**Aneurysm configuration**	**Neck width (mm)**	**Dome width (mm)**	**Dome height (mm)**	**Parent artery diameter (mm)**	**Treatment strategy**	**Device**	**Size**	**OKM after FD**	**1st FU (months)**	**OKM 1st FU**	**2nd FU (months)**	**OKM 2nd FU**
1	71	Distal BA	Fisher IV	Blister	0.6	0.8	1.1	2.4	Primary	1xp48_HPC	3 × 12	A3	4 months	B3	7.5 months	D1
2	38	A1 right	No	Saccular	3.2	5.8	4.5	2.1	Primary	1xp48_HPC	3 × 15	A3	3	D1	-	-
3	51	Distal BA	No	Saccular	8.2	35	25	2.5	Primary	2xp48_HPC	3 × 18	A3	4	B2	6.5	C2
4	77	M1/M2 right	No	Saccular	7	13	20	2.4	Plug and Pipe, pCANvas	1xp48_HPC	3 × 18	A3	3	B2	11.5	B2
5	63	A1/A2 left	No	Saccular	4	48	41	2.4	Plug and Pipe, pCONus	1xp48	3 × 18	A2	4.5	D1*	-	-
6	50	M1/M2 left	Fisher IV	Saccular	2	3.3	3.3	2	Plug and Pipe	1xp48_HPC	2 × 15	A1	3.5	D1	9.5	D1
7	53	M1/M2 right	No	Saccular	4.4	4.6	5	2.1	Plug and Pipe	1xp48	3 × 18	A1	3	B1	9.5	B1
8	60	M1/M2 right	No	Saccular	4.5	5.2	4.4	2.3	Primary	1xp48	3 × 15	A3	12.5	C1	-	-
9	49	A1/A2 left	Fisher IV (C6)	Saccular	1.7	2	1.5	1.8	Primary	1xp48_HPC	2 × 15	A2	4.5	D1	15.5	D1
10	41	M1/M2 right	Fisher IV	Saccular	7	10	3.7	2.9	Plug an Pipe	1xp48_HPC	3 × 18	A2	3	B2	8	B2
11	35	A1/A2 left	No	Saccular	3.6	6.2	3.7	3/2.5	Primary	1xp48	3 × 18	A2	1	A2	3.5	B2
12	51	Carotid- T	Fisher IV	Saccular	2	2.1	2.4	3.2	Primary	1xp48	3 × 18	A2	3	B3	9	B3
13	81	A1/A2 right	No	Saccular	4.7	5.8	4	2.5	Primary	1xp48_HPC	3x15	A1	3 months	B1	-	-
14	66	C6 right	No	Saccular	3.7	5.7	4.8	3.1	Primary	1xp48	3x18	A2	3 months	B1	9 months	B1
15	69	Distal BA	No	Fusiform	-	-	-	-	Primary	1xp48_HPC	3x18	A1	5 months	A1	7 months**	A1
16	64	BA/PCA right	Fisher IV	Saccular	1.7	2.4	2.3	2.3	Primary	1xp48_HPC	2x15	A3	3 months	B1	9 months	B2
17	69	RCP right	No	Saccular	3.6	5	4	1.8	Primary	1xp48_HPC	2x15	A3	3.5 months	D1	13 months	D1
18	71	C6 left	Fisher IV	Saccular	3.1	3.5	2.5	3.3	Primary	1xp48_HPC	3x18	A3	3.5 months	A3	11 months	A3
19	50	M1/M2 right	No	Saccular	4	14	13	2.3	Primary	1xp48_HPC	3x15	A3	4 months	A3	-	-
20	63	A1/A2 right	No	Saccular	4	48	41	1.8	Second look from the right	1xp48_HPC	2x25	B1	4 months	D1*	-	-
21***	66	Distal BA	No	Saccular	0.6 0.7	4.5 1.3	6 1.9	2.4	Primary	1xp48_HPC 1xp48_HPC	3 × 18 3 × 9	A3 A3	3.5 3.5	C2 A2	11 11	C2 A2
22	61	M1/M2 left	No	Saccular	3.5	4.2	5.8	2.3	Primary	1xp48_HPC	2 × 15	A3	9	D1	-	-
23	84	C7 left	Fisher IV	Blister	-	-	-	-	Primary	2xp48_HPC	3 × 18	A1	-	-	-	-
24	59	M1/M2 right	No	Saccular	5	6	5.5	2.7	Primary	1xp48_HPC	3 × 15	A1	3.5	A1	9	C1
25	47	VA right	Fisher IV	Dissecting aneurysm	-	-	-	-	Primary	4xp48_HPC 3xp48_HPC 1xp48_HPC	3 × 18 3 × 15 3 × 12	A3	-	-	-	-
26	47	A1/A2 right	No	Saccular	2.4	2.2	3.8	2	Primary	1xp48_HPC	2 × 15	D1	-	-	-	-
27	58	C7 left	No	Blister	0.8	06.-	1-	2.7-	Primary	1xp48_HPC	3 × 15	-	3	-	7.5	-
28	66	M1/M2 left	No	Saccular	4.7	7.6	6.5	2.8	Primary	1xp48_HPC	3 × 18	A3	3	A2	3	A2
29	42	V3/V4 left	No	Dissecting aneurysm: disconnection of previously implanted FDS	-	-	-	-	Revision	1xp48_HPC	3 × 18	-	-	-	-	-
30	69	C6 right	No	Saccular	6	7.1	5.1	3.5	Plug and pipe	3xp48_HPC	3 × 18	A3	3	A2	3	A2
31	48	BA tip	Fisher IV	Saccular	5	5.2	3	2.6	Plug and pipe	1xp48_HPC	3 × 18	A3	3	B1	9.5	D1
32	52	M1/M2 left	Fisher IV	Saccular	1.7	1.9	2.5	2	Plug and pipe	1xp48_HPC	3 × 12	A2	4	C1	-	-

* *Flow diversion remaining sufficient but de-novo perfusion from the contralateral right side required additional flow diverter implantation*.

** *Patients first angiographic follow-up revealed a hemodynamic relevant stenosis of the flow diverter requiring re-treatment. At second follow-up an additional balloon-expandable coronary stent was implanted. Five months later control-DSA revealed distinct decrease in aneurysm perfusion (OKM B1)*.

*** *The patient revealed two aneurysms at the distal basilar artery. After implantation of the first p48_HPC the aneurysm neck at the right SUCA was covered sufficiently. However, as proximal shortening of the device occurred a second device was mandatory for efficient flow diversion at the more proximal located lesion at the right AICA*.

In 26 cases, a single p48MW was used; and in four cases, implantation of two overlapping p48MWs was necessary to sufficiently cover the target aneurysm. In one case, 8x p48MWs in overlapping fashion were required to reconstruct a ruptured dissecting aneurysm of the V4 segment of the dominant vertebral artery. The dissection originated from the proximal V4 segment and extended into the proximal third of the basilar artery (BA). As a consequence of the length and the large changes in caliber, 8x p48MWs were necessary to reconstruct the dissected segment with sufficient overlap between the implanted devices. In another case, the reconstruction of a severely dysplastic ICA, harboring a broad based giant aneurysm in the ophthalmic segment and two further broad-based aneurysms originating from the supraclinoid segment required implantation of 3x p48MWs in telescoping technique.

Except for the treatment of a ruptured blister aneurysm originating from the C7 segment of the ICA and the reconstruction of the ruptured dissecting V4 aneurysm, all remaining cases were treated in an elective setting. Of those, eight treatments were performed as a second step after coiling (“plug and pipe”); 22 treatments were primary. There were two retreatments after prior flow diverter implantation, with unsatisfactory results.

Nine treatments were performed in the posterior circulation. The p48 was used for aneurysms arising from the distal BA bifurcation (basilar tip) in six patients; and in three patients, the p48 was implanted for treatment of aneurysms arising from the V4 segment of a dominant vertebral artery.

Twenty-three interventions were performed in the anterior circulation. Among those, 10 treatments were performed for aneurysms located at the middle cerebral artery (MCA) bifurcation, seven were used in the distal intracranial ICA, and five were used in the AcomA complex.

An overview of individual clinical and demographic information is provided in Table 1.

### p48MW vs. p48MW Hydrophilic Coating

In November 2018, a coated variant of the p48MW obtained CE mark and has been made available for European patients. The HPC has been tested extensively *in vitro* and *ex vivo*, and the first clinical experiences have been reported (16–21). Basically, the coating consists of a covalently bonded pentasaccharide layer at the surface of the device, which prevents platelet and leukocyte adhesion at the surface of the nitinol mesh. As a consequence, the p48MW HPC is less thrombogenic and facilitates significant reduction of platelet function inhibition without an increased risk of thromboembolic complications. Given the clinical outcomes after treatment with the HPC devices and the associated opportunity to reduce platelet-inhibitory medication, we later decided to exclusively implant the HPC variants of the p48MW after it had become available.

### Interventional Procedure

Informed consent for elective endovascular treatment was obtained from all patients. Oral dual platelet medication [500 mg of acetylic salicylic acid (ASA) and 180 mg of ticagrelor PO daily] was started the day before the procedure in elective cases. At the day of the procedure, the standard regimen, consisting of 100 mg of ASA once a day lifelong and 90 mg of ticagrelor (PO twice a day, for 12 months), was initiated.

In two cases of emergency treatments, 1,000 mg of ASA IV was administered, and sufficient mono-platelet inhibition was confirmed with Multiplate prior to treatment.

All endovascular procedures were performed under general anesthesia using a biplane angiography system (Philips AlluraClarity, Best, the Netherlands). Endovascular access was established *via* the right femoral artery using an 8-French introducer sheath (Terumo radifocus II, Leuven, Belgium). A bolus of heparin (5,000 IE) was administered *via* the sheath initially. A Neuron Max 088 (Penumbra, Alameda, USA) was used as guide catheter for supra-aortic extra-cranial access in combination with the Sofia distal access catheter (6F, 115 cm; MicroVention, Aliso Viejo, USA) for intracranial support. The recommended Prowler Select Plus (Cerenovus, Irvine, CA, USA) was used for delivery of the p48MW HPC in all cases. All procedures were performed by two each of three available neurointerventionalists with 5, 14, and 18 years of experience.

### Difficult Access—The Cast-pRESET Maneuver

In seven cases (4x AcomA, 1x MCA, 1x ICA C7, and 1x PICA/V4), an extraordinary level of supra-aortic and intracranial vessel elongation combined with unfavorable, acute-angled courses of the respective target vessel required a low-profile stent retriever-based exchange maneuver for introduction of the microcatheter essential for p48MW delivery. For this, the anatomically challenging target segment was catheterized with an atraumatic Excelsior SL 10 microcatheter (Stryker Neurovascular, Cork, Ireland). As a next step, in order to prevent distal wire dislocations and potentially critical vascular injuries related to the exchange maneuver, a low-profile stent retriever (pREset lite 3-20, phenox, Bochum, Germany) was transiently deployed in a secure location distal to the target segment. Using the pRESET lite as static substitute for the long and stiff exchange wire, the Prowler Select Plus delivery microcatheter was introduced along the pusher of the pREset lite, after the Excelsior SL 10 had been removed. This way, the Prowler Select Plus could be navigated to an otherwise unachievable endovascular position for p48MW delivery. An illustrated step-by-step protocol of the cast-PRESET maneuver was published recently and provides a detailed video of the technique (22).

## Results

### Technical Aspects—Delivery, Implantation, and Primary and Secondary Shortening

**Figure 1:**
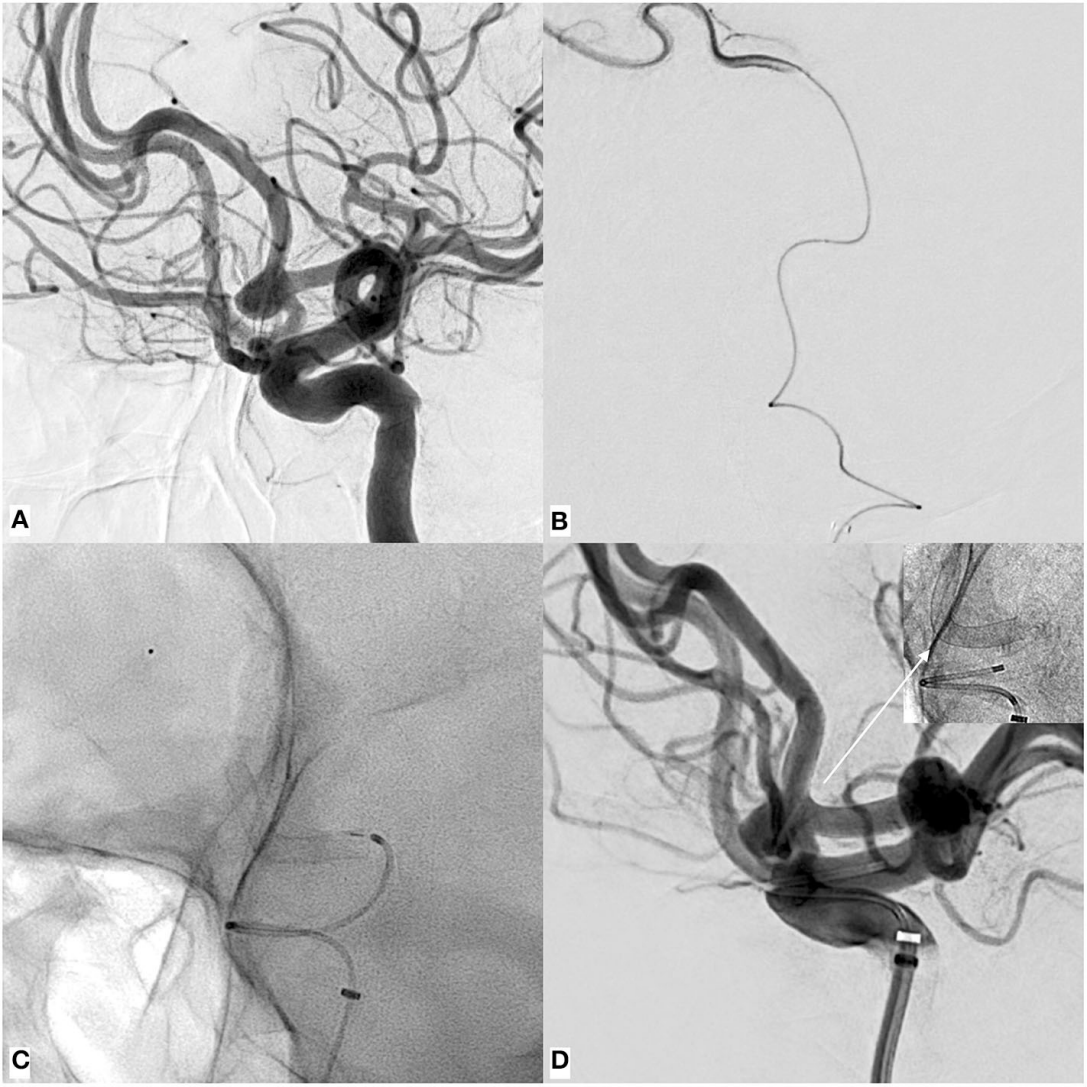
An example of p48MW hydrophilic coating (HPC) implantation for treatment of an un-ruptured, broad-based aneurysm of the AcomA complex *via* the left internal carotid artery (ICA). **(A)** Digital subtraction angiography (DSA) run in working projection revealing the broad based (neck: 5.3 mm), saccular aneurysm (fundus: 5.2 × 4.3 mm) predominantly filled by the left anterior cerebral artery (ACA). **(B)** Catheterization of the elongated, demanding ACA using the Excelsior SL 10 and confirmation of the correct endoluminal position distal to the aneurysm as a pre-requisite for safe microcatheter exchange/introduction of the Prowler Select Plus required for device delivery. **(C)** Plain radiography in working projection demonstrating flow diverter stent (FDS) implantation—note the radiopaque olive tip at the distal end of the p48 wire at the level of the distal A2 segment, which helped to stabilize the equipment during deployment. Implantation of the device has caused significant straightening of the A1–A2 transition, additionally contributing to the redirection of blood flow away from the aneurysmal orifice. **(D)** Control injection after implantation shows the patency of the vessel in combination with the location of the implanted device (radiogram upper right corner).

**Figure 2:**
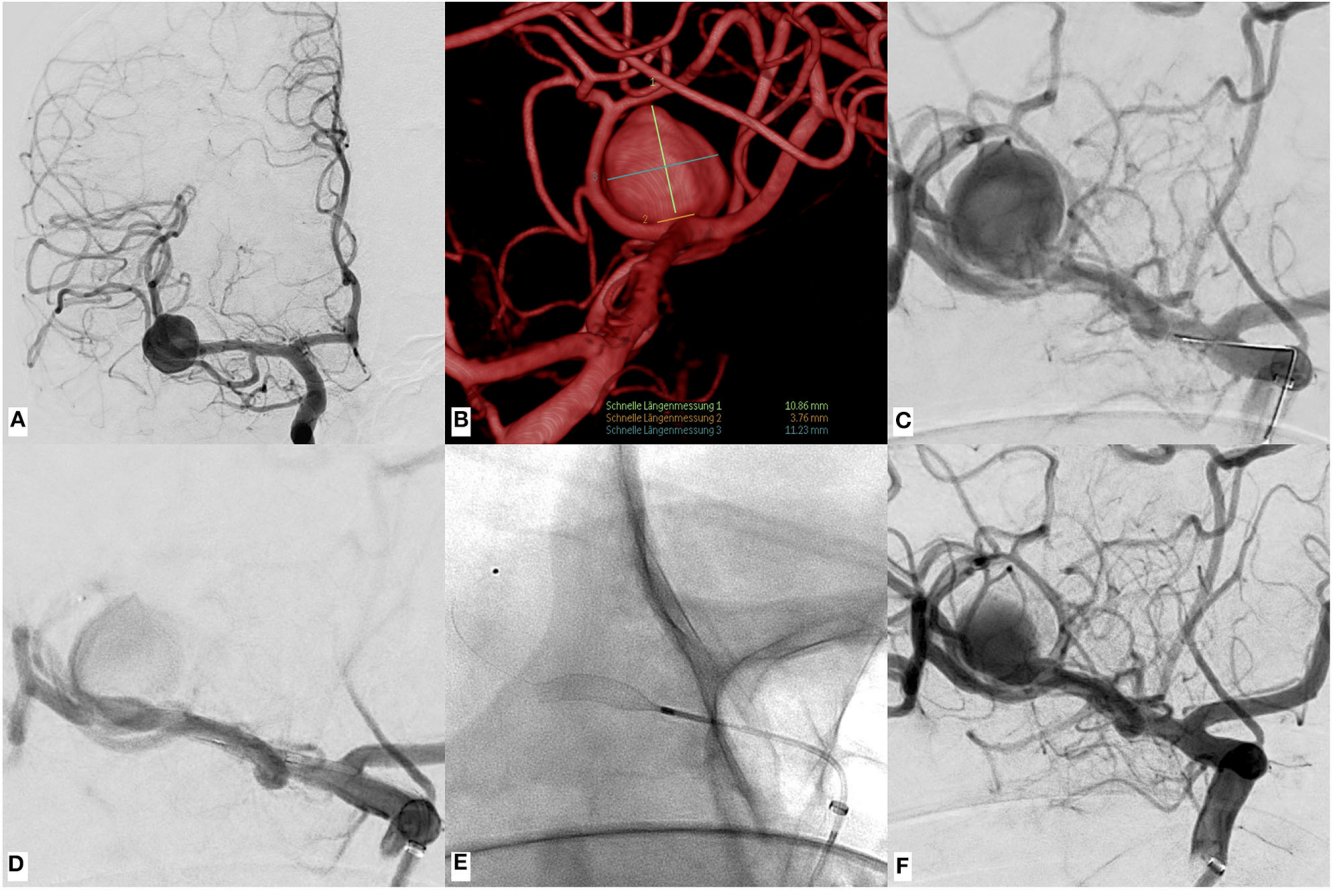
An example of p48MW hydrophilic coating (HPC) implantation in the right M1–M2 junction for treatment of an un-ruptured saccular aneurysm arising from the right middle cerebral artery (MCA) bifurcation. **(A)** Digital subtraction angiography (DSA) run in standard AP projection showing the large, laterally developed saccular MCA aneurysm. **(B)** Volume rendered reconstruction of the rotational 3D angiography revealing the relationship of the aneurysm to the associated MCA branches as well as the lesion dimensions. **(C)** Pre-intervention DSA run in working projection. **(D)** Control injection in working projection after definition of the distal landing zone. **(E)** Corresponding non-subtracted device radiogram. Note the position of the MW in the M2 segment, additionally stabilizing the device-microcatheter unit during deployment. **(F)** Final DSA run after p48MW HPC implantation demonstrates significantly reduced arterial filling compared with the initial diagnostic DSA (O'Kelly Marotta A3).

In all cases, the deployment of the FDSs was unremarkable. No device was lost within the microcatheter, and the transition from the hemostatic valve to the microcatheter went smoothly during all procedures. Figure 1 shows p48MW implantation for treatment of an AcomA aneurysm *via* the left ICA. Figure 2 demonstrates p48MW implantation for treatment of a MCA aneurysm of the right-handed side.

In three cases (2x basilar tip and 1x carotid artery bifurcation), significant distal shortening manifested either during or immediately after deployment, requiring the implantation of an additional p48MW to sufficiently cover the target aneurysm. Each shortening that occurred is related to the increased difficulty in defining the optimal distal landing zone, which will be explained briefly below. The distal basilar bifurcation with both proximal P1 segments and the carotid bifurcation including the proximal M1 segment gave rise to numerous important, perfusing non-compensable perforating branches. Additionally, the junction of the posterior communicating artery with the posterior cerebral artery (PCA), which should be avoided when implanting FDSs, lies comparatively proximal in a number of cases.

The BA/PCA junction and the ICA/MCA transition zone give rise to a number of important perforating arteries. As those perforators are not collateralizable terminal brain vessels, covering their origin with a flow diverter distinctly increases the risk for stroke. In order to reduce the risk for perforator infarction after flow diverter implantation to a minimum, application of a flow diverter device with only a moderate surface coverage—and therefore a higher porosity that facilitates permanent patency of terminal branches—is desirable. Thus, for the choice for the p48MW, however, in retrospect, the distal landing zone of the p48 in the PCA was too short.

As a consequence of the short landing zone of the p48MW along the proximal P1 segment combined with the significant increase in caliber that naturally occurs at the transition from PCA to BA and the almost 90° angle between those segments, caused the p48MW to shorten distally and deploy completely in the BA.

**Figure 3:**
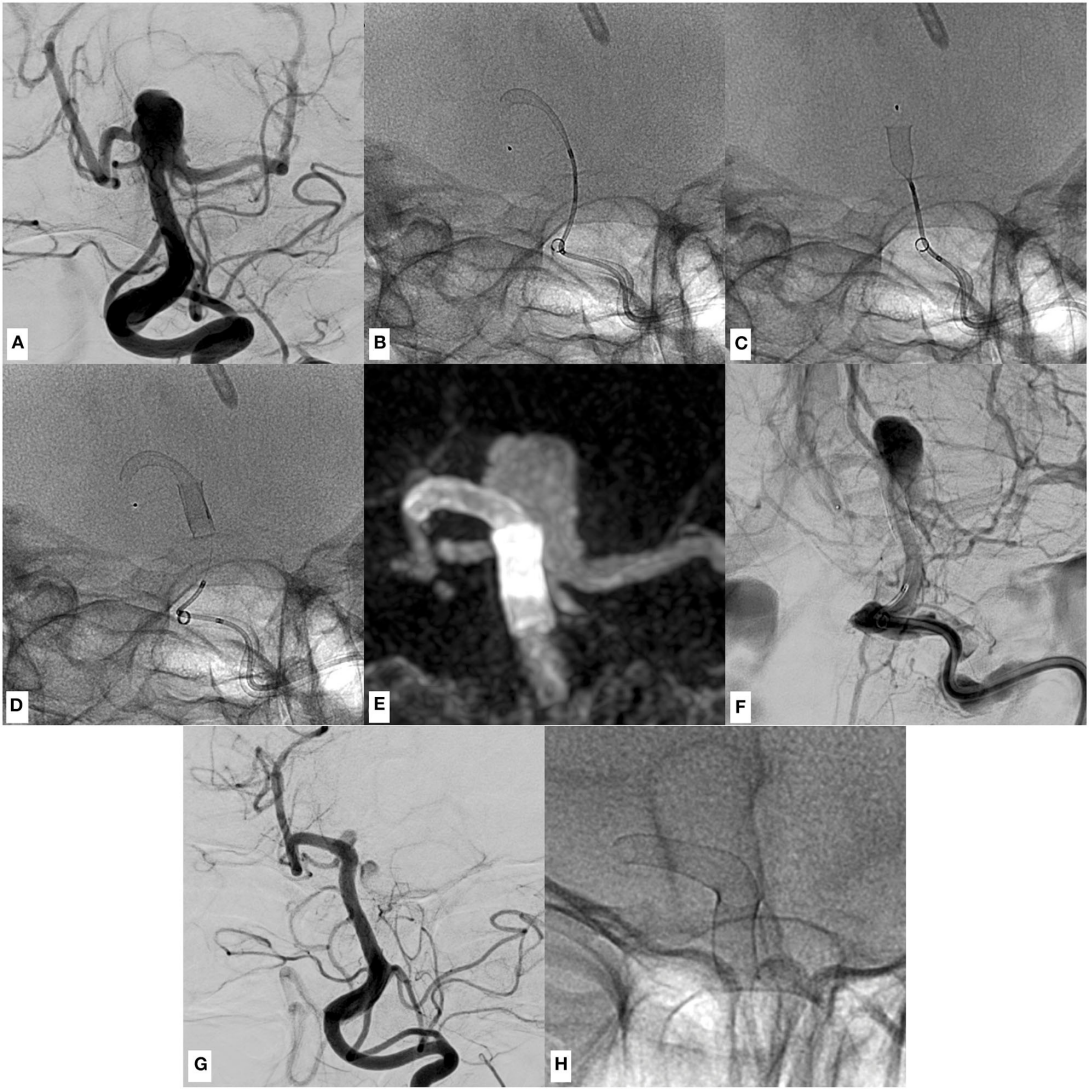
An example of p48MW hydrophilic coating (HPC) implantation in the right posterior cerebral–basilar artery (BA) junction for treatment of a broad-based, partially thrombosed, un-ruptured aneurysm arising from the basilar tip, causing progressive hydrocephalus. **(A)** Conventional angiogram in working projection shows the perfused part of the saccular, broad-based aneurysm of the BA–posterior cerebral artery (PCA) complex (neck, 7 mm; fundus, 7 × 8 mm). **(B)** Positioning of the first p48MW HPC. The distal landing zone, aiming to cover the smallest perforator-rich area as possible, was defined as comparatively short. Note the distal end of the movable wire (radiopaque olive) within the distal P1 segment, which was used to further stabilize the system during implantation. In retrospect, a more distal positioning of the MW in the middle part of the P2 segment may have prevented dislocation of the first device, as demonstrated in the following images. **(C)** Simultaneous to the deployment of the device, significant distal shortening and subsequential proximal dislocation of the entire device occurred, requiring the implantation of a second p48MW HPC in order to sufficiently cover the broad aneurysmal orifice. **(D)** Device radiogram showing the second p48 implanted in telescoping technique. Now the aneurysm is covered sufficiently. Initially, wall apposition/flow diverter opening appeared to be only moderate, which resolved a few minutes after implantation without the necessity for further manipulation. **(E)** Maximum intensity projection of the flat-panel CT shows the flow diverter stent (FDS) construct and its relation to the BA–PCA junction and the broad-based aneurysm. **(F)** Venous phase of a digital subtraction angiography (DSA) run in working projection after successful implantation shows protracted opacification of the aneurysm sac (O'Kelly Marotta: A3) indicating the extent of the flow diversion effect. **(G)** Follow-up DSA in working projection 3 months later shows the remodeled posterior circulation with a minimally perfused remnant of the aneurysm. **(H)** Device radiography in corresponding projection.

In this situation, where distal shortening and proximal dislocation of the p48MW occurred, the distally placed movable wire (MW) enabled re-positioning of the Prowler Select Plus into the distal target segment, allowing for implantation of a second p48MW (Figures 3A–H show an example of distal shortening + proximal dislocation and its successful management in case of an aneurysm arising from the tip of the basilar artery).

### Shortening in Case of Undersizing

Related to the clinically severe case (Hunt & Hess 4, Fisher 3) of a ruptured dissecting aneurysm of the dominant vertebral artery, interdisciplinary consent was made for reconstruction of the dissected segment with the p48MW HPC. By using this device, we accounted for the potential necessity of reduced platelet-inhibitory medication in case the patient needed additional neurosurgery, for example, ventricular shunt placement or decompressive craniectomy. However, the largest version of the p48MW HPC available is the 3 mm × 18 mm (3–18) variant, and the V4 segment exhibited diameters ranging from 3.0 to 3.8 mm. As a consequence, significant undersizing was unavoidable. In case of undersizing, the 3–18 mm device can gain a maximal diameter of 3.8 mm but then shortens longitudinally to ~5 mm. As the dissecting aneurysm extended along the whole V4 segment, telescoping of 8x 3–18 p48MW HPC was necessary to sufficiently reconstruct the dissected segment.

### Initial Non-opening of the Midsection After Deployment

In three cases (1x distal BA bifurcation, 1x MCA bifurcation, and 1x AcomA complex), incomplete unfolding of the adequately sized FDSs required balloon angioplasty immediately after implantation. Those three cases were associated with significant tortuosity of the respective landing zones. In all of those cases, a Scepter C balloon modeling catheter (MicroVention, Aliso Viejo, USA) was used to create optimal opening and wall apposition of the device.

### Mean Fluoroscopy Times Grouped According the Aneurysm Location

As a surrogate marker for overall complexity of the individual endovascular treatments, fluoroscopy times of the interventions were also reviewed. In brief, the following results (means and standard deviations) were obtained: AcomA complex, 33.5 ± 20.2 min; MCA bifurcation, 25.1 ± 12.3 min; C6–C7 segment of the ICA, 49.7 ± 44 min; BA–PCA complex, 24.7 ± 12.3 min; and V3–V4 segment of the vertebral artery, 79.7 ± 65.5 min.

### Outcome and Follow-Ups

Our routine follow-up regimen after FDS implantation includes an unenhanced cranial CT 24–48 h after implantation and follow-up digital subtraction angiographies (DSAs) 3, 9, and 24 months post-procedure. In all patients, the cranial CT was unremarkable. As of now, 14 patients had the first follow-up DSA; post-procedural flow diverter efficacy and 3 months' results, using the O'Kelly Marotta scale (23), are presented in Table 1.

One patient treated for disconnection of two previously implanted flow diverters in the V3–V4 segment (initial treatment for reconstruction of a dissecting aneurysm of the dominant vertebral artery) suffered a peri-procedural parenchymal hemorrhage in context of a critical hypertensive episode under dual platelet inhibition and transient anticoagulation with heparin, which necessitated craniotomy and surgical evacuation of the hematoma. The patient who was treated primarily for a ruptured dissecting aneurysm of the right-handed side dominant vertebral artery suffered a periprocedural, thromboembolic occlusion of the left PCA resulting in the infarction of the PCA territory. Otherwise, no procedural or post-procedural complications occurred in our cohort.

## Discussion

This study summarizes our initial experiences with the use of the novel low-profile p48MW flow diverter for treatment of cerebral aneurysms localized in the vertebrobasilar territory and the anterior circulation distal to the superior hypophyseal artery segment.

Compared with conventional large-bore microcatheters necessary for application of most second-generation FDSs [for example PED2 (Medtronic/USA), DERIVO (acandis/Germany), and Surpass (Stryker/USA), which all warrant a 0.027 in. inner diameter microcatheter], the p48MW only requires a 0.021 in. inner diameter microcatheter for deployment. This feature considerably simplifies treatment of aneurysms in more peripheral segments beyond the circle of Willis, as the 0.021 in. Prowler Select Plus facilitates atraumatic access of the comparatively tortuous post-bifurcation arteries. Nevertheless, in a minority of our cases, primary catheterization even with the versatile Prowler Select Plus was impossible. In those situations, initial access with a lower-profile microcatheter—the Excelsior SL 10—was required to enable the above exchange maneuver, which eventually allowed placement of the Prowler Select Plus in a position sufficiently distal to the origin of the aneurysm. After the endovascular position that guaranteed enough forerun for unimpeded advancement of the FDSs was obtained, the proximal support of the Prowler Select Plus was adequate for precise deployment in all cases.

Unique to the p48MW is a MW, which can be advanced independently and used as an “anchor” at a position up to 60 mm distal to the proximal end of the device. This way, precise definition of the distal landing zone, particularly tortuous target segments (for example, the M1–M2 transition, the BA–PCA transition, and most importantly the A1–A2 junction), is facilitated; and sudden proximal dislocations of the FDS microcatheter unit are reduced to a minimum. Furthermore, the MW can be kept in a stable position until the FDSs is fully implanted. As a consequence, re-access of the treated vessel distal to the implanted FDSs using the available delivery microcatheter *in situ*—without losing the potentially very difficult distal endovascular access—becomes easily feasible and allows uninterrupted implantation of a second p48, if necessary.

However, shortening of the p48MW has emerged as a significant and frequent shortcoming of the device, which reliably occurred if the device was deployed over transition zones associated with large, sudden changes in luminal diameter or if undersizing was unavoidable. The most impressive case in this regard was the singular patient in our cohort who required immediate treatment for a ruptured dissecting aneurysm of the dominant vertebral artery. The decision for reconstruction with the p48MW HPC was made for two reasons: first of all, catheterization with a 0.027 in. microcatheter proved technically impossible without risking further considerable injury of the fragile segment, which may have culminated in critical re-hemorrhage. Secondly, the p48MW HPC is the only low-profile flow diverter that allows application of a single antiplatelet function medication in conditions that expectably require further surgery (11, 17, 18), although class 3 evidence is still lacking in this regard. Eventually, a total of 8x p48MW HPC 300 (300-18, 4x; 300-15, 3x; and 300-12, 1x) were implanted using telescoping technique to reconstruct the segment with extensive mural injury. The latter exhibited a proximal diameter of 3.8 mm, a distal diameter of 2.6 mm, and a length of ~28 mm. Well corresponding to the information provided in the manual of the device, application of the p48MW HPC 300-18 within a vessel showing a luminal diameter of 3.8 mm resulted in >70% shortening, which explains the high number of devices required for the reconstruction of this rather short segment.

The p48MW HPC offers a unique constellation of features as a flow diverter, but its safe application in demanding cases, such as ruptured dissecting aneurysms and challenging individual vascular anatomies, requires substantial experience of the endovascular surgeon with this device.

Currently, the Silk Vista Baby (Balt, France) is the only alternative low-profile FDS available for treatment of small cerebral vessels (diameters from 2.25 to 3.25 mm) (13). The Silk Vista Baby is implanted *via* a 0.017 in. microcatheter, which can be advantageous for extremely tortuous intracranial vessels, in comparison with the p48MW, which requires a 0.021 in. microcatheter. However, the MW of the p48MW is a helpful distinct feature that allows greater stability of the microcatheter device unit during the process of implantation. Furthermore, there is no coated version of the Silk Vista Baby, allowing reduction of antiplatelet function treatment. Comparable reports on the efficacy and intermediate or long-term outcomes after implantation of each of the devices are lacking.

As a consequence, both devices offer specific advantages for the treatment of small cerebral vessels and can be considered complementary. Further studies are required, especially reporting intermediate and long-term outcomes.

## Conclusions

In summary, our yet limited experience and short-term follow-up results indicate a distinct aptitude of the device for endovascular therapy of distal bifurcation aneurysms. A total of 16 aneurysms originating from the AcomA complex and the MCA bifurcation were treated uneventfully without encountering any procedure-related or post-procedural complications. Furthermore, six aneurysms originating from the distal BA or the proximal P1 segment, requiring the coverage of the BA–PCA junction, were treated equally successfully. Considering the significant number of perforating arteries arising from these segments (24) in context of previous investigations that reported a mean procedure-related mortality rate of ~15% (25) and symptomatic ischemic stroke rates as high as 18% (26–28), the p48MW potentially provides increased safety for endovascular treatment of complex bifurcation aneurysms (29).

Our study suffers from a number of limitations. Experiences with the device presented in this manuscript were only gathered in a single institution with a comparatively small collective of patients. Additionally, only procedural and short-term outcomes are available, which corroborates the necessity of further studies of midterm and long-term outcomes. A generalized statement on the feasibility of the hydrophilically coated p48MW for treatment of ruptured aneurysms in context of reduced pharmaceutical platelet function inhibition on the basis of our results is impossible. Further studies are required to corroborate our experience in this regard.

## Data Availability Statement

All datasets generated for this study are included in the article/supplementary material.

## Ethics Statement

Our study—systematically reviewing cases of p48MW implantations starting in January 2019—was approved by the institutional ethics committee (local IRB nr. AZ 208-15-0010062015). Informed consent of each patient regarding the scientific use of radiological and clinical data was obtained in writing either from the patient himself or his/her legal representative.

## Author Contributions

SS and MK: contributed equally to the paper. UQ: performed the interventions. KH: wrote and reviewed the paper. M-SS: performed image analysis and statistical review. SM: performed review of the clinical information and corrected the paper. JM: performed image analysis and wrote the paper. CS: performed interventions and follow up imaging and analysis. CR: performed interventions and post-processed imaging data. MK: performed image analysis, proofread and corrected the paper, and performed statistical analysis. SS: designed the study, wrote the paper, and performed interventions. All authors contributed to the article and approved the submitted version.

## Conflict of Interest

The authors declare that the research was conducted in the absence of any commercial or financial relationships that could be construed as a potential conflict of interest. The reviewer PB declared a past collaboration with one of the authors SS to the handling editor.

## Abbreviations

ACA: anterior cerebral artery
AcomA: anterior communicating artery
BA: basilar artery
FDS: flow diverter stent
HPC: hydrophilic coating
ICA: internal carotid artery
MCA: middle cerebral artery
PCA: posterior cerebral artery
PcomA: posterior communicating artery.
